# Outcome Following Open Repair of Hereditary and Non-Hereditary Thoracoabdominal Aortic Aneurysm in Patients Under 60 Years Old—A Multicenter Study

**DOI:** 10.3390/jcm14072513

**Published:** 2025-04-07

**Authors:** Jelle Frankort, Siebe Frankort, Panagiotis Doukas, Christian Uhl, Michael J. Jacobs, Barend M. E. Mees, Alexander Gombert

**Affiliations:** 1 Department of Vascular Surgery, Medical Faculty, RWTH Aachen University, 52074 Aachen, Germanyagombert@ukaachen.de (A.G.); 2 Department of Vascular Surgery, MUMC+ Maastricht, 6229 HX Maastricht, The Netherlands; 3 Institute of Statistics Netherlands (CBS), 6401 CZ Heerlen, The Netherlands

**Keywords:** thoracoabdominal aortic aneurysms, open surgical repair, hereditary aortopathy

## Abstract

**Objective:** This multicenter study compares outcomes of open thoracoabdominal aortic aneurysm (TAAA) repair in patients < 60 years with (n = 106), versus without (n = 167), hereditary aortopathy. **Methods:** We conducted a retrospective analysis of 273 consecutive open TAAA repairs (2000–2024) at two European centers. The primary endpoint was early outcome. We used a Kaplan–Meier curve to assess survival, and logistic regression to identify predictors. **Results:** Operative death rates were similar (hereditary: 13/106 [12.3%] vs. non-hereditary: 22/167 [13.2%], *p* = 0.83). Hereditary aortopathy patients were younger (median 42 vs. 54 years, *p* < 0.001) with lower BMI (24.1 vs. 28.4 kg/m^2^, *p* < 0.001). Non-genetic patients had higher rates of chronic kidney insufficiency (58/167 (34.7%) vs. 14/106 (13.2%), *p* < 0.001), coronary artery disease (43/167 (25.7%) vs. 9/106 (8.5%), *p* < 0.001), and prior myocardial infarction (31/167 (18.6%) vs. 4/106 (3.8%), *p* < 0.001). Hereditary aortopathy patients suffered more often from post-dissection TAAA (68/106 [64.2%] vs. 44/167 [26.3%], *p* < 0.001) and prior aortic surgery (81/106 (76.4%) vs. 79/167 (47.3%), *p* < 0.001). Pulmonary complications (67.0% vs. 61.1%, *p* = 0.32), acute kidney injury (25.5% vs. 22.8%, *p* = 0.61), and spinal cord ischemia (6.6% vs. 10.2%, *p* = 0.31) were comparable between groups. Overall 5-year survival was 65.7%; the rate of any reintervention during follow up was 21.2%. Logistic regression identified no predictors for perioperative mortality. **Conclusions:** Open TAAA repair in patients < 60 years carries relevant perioperative mortality, which is comparable between hereditary and non-hereditary groups; non-hereditary patients had impaired preoperative cardiopulmonary status.

## 1. Introduction

Thoracoabdominal aortic aneurysms are a complex vascular pathology that carry substantial risks due to their vulnerability to rupture and the highly intricate surgical interventions necessitated for their management [1,2]. While open surgical repair has traditionally been the gold standard, endovascular repairs have become the standard of treatment for most patients suffering from aortic aneurysm, particularly for older and higher-risk patients, as related complications and mid-term results are favorable [3,4]. Current guidelines from the European Society for Vascular Surgery (ESVS) and the American Heart Association (AHA) continue to recommend open surgical repair for TAAAs in fit patients or those with hereditary aortopathies [5,6,7], particularly in younger patients where long-term durability is paramount.

However, current guidelines on thoracoabdominal aortic aneurysm repair have been challenged, as some studies suggest that endovascular repair may be a viable alternative for young and fit patients [3,8]. Due to improvements in endovascular grafts, patients previously unsuitable for endovascular repair, such as those with post-dissection aneurysms or hereditary aortopathy patients, are increasingly being considered for endovascular repair. The recent EVICTUS study demonstrates promising results, reporting a 5-year survival rate of 75% in connective tissue disease patients who underwent thoracic endovascular aortic repair; however, the reintervention rate remains relatively high [9].

The specific outcomes of open TAAA repair in young patients with hereditary versus non-hereditary aortopathies remain unclear. This comparison is particularly important as young patients with hereditary aortopathies are routinely recommended for open repair despite the evolution of endovascular techniques. Recent results on open TAAA repair are scarce and cohort sizes are small in young patients. Di Luozzo et al. (2013) reported an in-hospital mortality rate of 4.7% in young patients undergoing open TAAA repair [10]. A Japanese study from 2019, focusing on TAAA repair in patients younger than 50 years old, found exceptional results with a mortality rate of 3%, but only included 100 patients [11]. Our study aims to address this knowledge gap by comparing the outcomes of open TAAA repair in patients under 60 years old with and without hereditary aortopathies. Understanding whether young patients with hereditary aortopathies have different outcomes compared to their non-hereditary counterparts is crucial for optimizing treatment recommendations and potentially refining the current guidelines that recommend open repair for all hereditary aortopathy patients. This retrospective study analyzed the outcomes and follow up of open thoracoabdominal aortic aneurysm repair in patients 60 years or younger and in those patients with a hereditary aortopathy treated at our institution, in both Maastricht, NL, and Aachen, DE, between 2000 and 2024.

## 2. Materials and Methods

In this retrospective study, we analyzed the outcomes of open thoracoabdominal aortic aneurysm repair in patients of under 60 years old, with or without hereditary TAAA. This study, approved by the local internal review board (EK004/14, approval date 20 April 2020), was reported according to the STROBE criteria [12].

This cross-border study was conducted in two university hospitals in Maastricht, the Netherlands, and Aachen, Germany. Both centers are designated as reference centers for open TAAA repair. The study population was identified from our institutional database of patients who underwent open thoracoabdominal aortic aneurysm repair between January 2000 and December 2023. Detailed clinical data were collected, including patient demographics, comorbidities, aneurysm characteristics, and surgical details, as well as several outcome parameters.

Inclusion criteria were elective or emergency open repair for thoracoabdominal aortic aneurysm (TAAA), categorized by the Crawford classification in patients younger than 60 years old. The definitive treatment plan was made by a multidisciplinary team involving vascular surgeons, cardiac surgeons, interventional radiologists, and anesthesiologists. Younger patients with hereditary TAAA were primarily managed through open repair.

### 2.1. Surgical Protocol

The surgical protocol employed at our institution during the study period has been previously described in detail [13,14]. Briefly, the protocol included selective double-lumen tube intubation with cerebrospinal fluid drainage and the perioperative monitoring of motor-evoked potentials. The patient is positioned on a beanbag in a modified right lateral decubitus posture, while elongating the operating table to facilitate optimal access to the thoracic cavity, sequential aortic clamping, femoro-femoral extracorporeal circulation with distal aortic perfusion, selective visceral perfusion, and mild hypothermia (32–33 °C). The surgical protocol and incision were tailored to the type and extent of aneurysm.

### 2.2. Definitions

Emergency repair was defined as treatment within 24 h because of symptomatic or ruptured TAAA. Chronic kidney disease was defined as a preoperative estimated glomerular filtration rate less than 60 mL/min/1.73 m^2^. Acute kidney injury (AKI) within 48 h postoperatively was defined according to the Kidney Disease Improving Global Outcomes (KDIGOs) criteria based on serum creatinine levels [15]. Operative death was defined as any death within 30 days of surgery or during hospital stay. Lung complications included pneumonia, prolonged artificial ventilation necessitating a tracheotomy, and acute respiratory distress syndrome [16,17]. Any surgical reintervention after hospital admission was defined as reoperation. Sepsis and shock were defined according to current guidelines [18,19]. Cardiac complications included myocardial infarction, acute heart failure, and ventricular tachycardia. Massive transfusion was defined as the intraoperative administration of ≥10 units of packed red blood cells.

### 2.3. Statistical Analysis

Data analysis was performed using RStudio (Version number: 4.4.0, R Core Team, PBC, Boston, MA, USA) and packages available through the package repository CRAN. All data are presented as median with the range or number of patients (n) and their relative amount (%). Comparisons between overall groups and those with and without hereditary aortopathy were conducted via Chi test for variables with number of patients or Mann–Whitney U test for variables with median. To investigate the relationship between operative mortality and the significant variables, a binary logistic regression analysis was conducted. Analysis differences were assumed to be significant with *p* < 0.05. The Hosmer–Lemeshow test was used to assess the goodness of fit of the multivariable logistic regression model.

## 3. Results

A total of 577 patients underwent surgical treatment for TAAAs between the years 2000 and 2024. Of the total number of patients, 273 were under the age of 60, while 106 patients (38.9%) were found to have a hereditary TAAA.

Distinct demographic differences emerged between groups, with non-hereditary TAAA patients being predominantly male, significantly older, and having higher BMI compared to hereditary TAAA patients (Table 1). The non-hereditary group also demonstrated a substantially higher burden of cardiovascular comorbidities, including chronic kidney disease, COPD, diabetes mellitus, arterial hypertension, coronary artery disease, and prior myocardial infarction (Table 1).

In contrast, hereditary TAAA patients showed characteristic disease patterns with a notably higher prevalence of post-dissection aneurysms and previous aortic surgical interventions (Table 1).

Marfan syndrome was the most frequent entity (90/106, 84.9%), followed by Loeys–Dietz syndrome (7/106, 6.6%) and vascular Ehlers–Danlos syndrome (3/106, 2.8%). Six cases (5.7%) were unspecified connective tissue disorders (Table 2).

Perioperative analysis revealed comparable surgical parameters between groups, with similar procedure times, rates of massive transfusion, and frequencies of emergency repairs (Table 3).

Most importantly, perioperative mortality showed no significant difference between hereditary (12.3%) and non-hereditary (13.2%) TAAA patients. Other postoperative outcomes were also comparable between groups, with similar rates of major complications including pulmonary complications, acute kidney injury, and spinal cord ischemia (Table 4). This suggests that, despite the different demographic and clinical profiles, both patient groups face similar perioperative risks.

**Figure 1:**
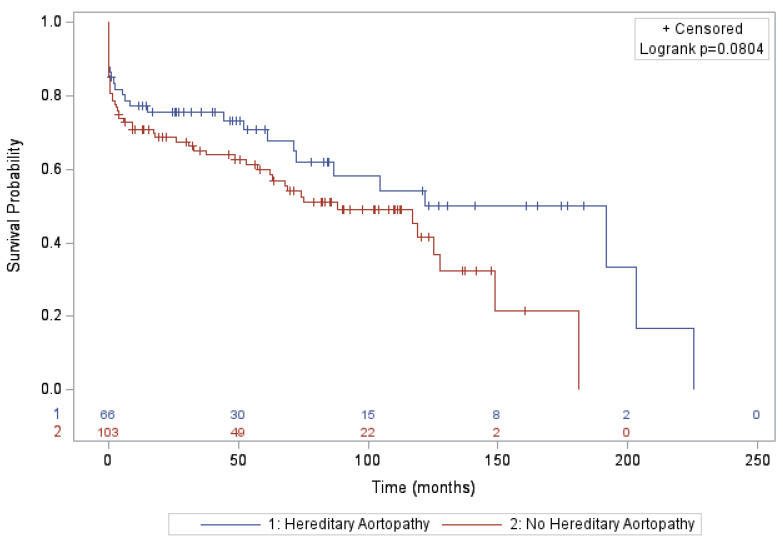
Analyses of survival. Kaplan–Meier curve depicting survival over a period after surgery (n = 170). Patients who were alive at the last follow up are indicated with tick marks along the curve.

Follow up could be completed for 170 of the 273 (62.3%) patients. A Kaplan–Meier curve depicting the survival rate for both groups can be found in Figure 1 (Survival analysis demonstrated no significant intergroup difference (log-rank *p* = 0.62). The aortic reintervention rate after discharge was 18/85 (21.2%).

A binary logistic regression analysis was performed to identify independent predictors of in-hospital mortality. After adjusting for potential confounders, including age, BMI, presence of hereditary aortopathy, and comorbidities, no single factor emerged as a statistically significant independent predictor of mortality. The complete results of this analysis are presented in Supplementary Table S1. The multivariable logistic regression model demonstrated adequate fit according to the Hosmer–Lemeshow test (χ^2^ = 24.062, *p* = 0.15), indicating no significant departure from model assumptions.

Subgroup analyses were performed to evaluate potential interactions between hereditary status and key clinical factors. In patients with pre-existing renal insufficiency, the development of postoperative acute kidney injury showed similar rates between hereditary and non-hereditary groups (27.3% vs. 31.4%, *p* = 0.61) (Supplementary Table S2). Likewise, among patients with previous aortic surgery, postoperative complication rates and mortality were comparable between hereditary and non-hereditary TAAA patients across all major complications including pulmonary (66.7% vs. 61.5%, *p* = 0.52), renal (26.5% vs. 22.8%, *p* = 0.60), and neurological (6.2% vs. 10.5%, *p* = 0.31) complications (Supplementary Table S3).

## 4. Discussion

This multicenter study of 273 open TAAA repairs in patients < 60 years reveals that hereditary TAAA patients, despite presenting with a notably higher prevalence of post-dissection aneurysms and previous aortic surgical interventions, achieved equivalent perioperative outcomes to non-genetic peers. Non-genetic patients’ higher comorbidity burden did not cause increased mortality- or morbidity rates.

This suggests that, although younger patients receiving open surgery because of non-hereditary TAAA presented with cardio-vascular morbidity at baseline, outcomes were comparable in both groups. This could indicate that the presence of a hereditary TAAA, such as Marfan syndrome or Ehlers–Danlos syndrome, may independently confer a risk. This counterintuitive result warrants careful consideration and may be explained by several factors. One possible explanation is that the chronic nature of hereditary TAAA may lead to more complex aortic pathologies and technically more challenging repairs. Also, we found that patients with a hereditary TAAA have a higher rate of post-dissection TAAA and more often have previous aortic operations, e.g., ascending repair in cases of type-a aortic dissection [20]. These two findings are comparable to other studies reporting on open TAAA repair and previous aortic operations [21,22,23,24].

The overall high rate of pulmonary complications in both the subgroups is particularly noteworthy, given that hereditary TAAA patients often have underlying lung involvement or differences in chest wall mechanics, as well as previous aortic repairs [25,26].

Also, the fact that there are no significant differences in postoperative outcome contrasts with other studies. For instance, Rebello et al. found that hereditary aortopathy patients had even better outcomes than non-hereditary aortopathy patients in open TAAA extent I repair. However, their study included patients of all ages, which may account for the difference in findings [27].

Further comparison between our results and those of other studies is difficult due the scarcity of data and cohort sizes in young patients. There are also no recently published studies on open TAAA repair in young patients. Older studies focusing on smaller cohorts of patients receiving open TAAA repair reported similar outcomes. However, some aspects can be compared. Di Luozzo et al. (2013) reported an in-hospital mortality rate of 4.7% in young patients undergoing open TAAA repair [10]. A Japanese study from 2019, focusing on TAAA repair in patients younger than 50 years old, found exceptional results with a mortality rate of 3%, but only included 100 patients [11]. A large US study comparing younger patients with older patients found better early outcomes in the younger patient cohort. However, this study did not compare outcomes between genetic and non-genetic patients within the younger group [28].

The trend towards endovascular repair, even in young and fit patients, is ongoing. A recent study on fenestrated and branched endovascular aneurysm repair (F/BEVAR) in younger patients found excellent short-term outcomes with favourable overall survival rates and lower perioperative mortality [29]. This highlights the need for studies comparing open and endovascular approaches in this particular subgroup of patients. It is important to note that while endovascular techniques offer potential benefits, they also come with specific concerns for younger patients. The long-term durability of endovascular repairs remains uncertain in younger patients, particularly in the context of hereditary aortopathies [8]. In younger patients with longer life expectancies, the likelihood of developing endoleaks and long-term failure of endovascular aortic repair must be carefully considered given the risks of late aneurysm rupture and the need for reintervention in patients. The retrospective, worldwide EVICTUS study described TAAA repairs performed in hereditary aortic aneurysm patients and showed promising results with a high rate of early technical success, low perioperative mortality, and a mid-term survival rate comparable with reports of open aortic surgery in patients with hereditary aortopathy, even though the reintervention rate was high [9]. Also, in post-dissection patients, the reintervention rate is considerable at 66% [8]. Our study demonstrated a similar five-year survival rate of 65%, yet achieved a significantly lower reintervention rate after discharge at 21.2%, suggesting potential advantages of open repair in terms of long-term durability. The reduced long-term survival observed in both endovascular and open TAAA repair patients may reflect the inherent high-risk nature of this patient population. A recent study by Rocha et al. confirmed that the long-term survival rate following TAAA repair remains significantly lower compared to the general population, regardless of being treated with endovascular or open techniques. This is probably due to the associated risk factors and cardiopulmonary status of patients with a TAAA [30].

The results of our study may have important clinical implications. In contrast with previous assumptions, the presence of hereditary TAAA and the concomitant higher incidence of previous aortic aneurysm repair and post-dissection aneurysms should be assessed as risk factors when planning TAAA repair in young patients, yet the impact on outcomes may be overestimated. However, all patients, regardless of genetic status, require careful preoperative optimization and postoperative monitoring, since the mortality and morbidity rates are still considerable.

Our findings also contribute to the growing body of knowledge on hereditary TAAA, complementing large-scale studies like the GenTAC Registry, providing data on the natural history and long-term outcomes of various genetic aortopathies. Our observation of higher rates of previous aortic surgery and aortic dissection in the hereditary TAAA group is consistent with GenTAC’s findings across various genetic conditions [31,32].

This study has several limitations. Its retrospective nature introduces potential for patient selection bias, confounding factors, and issues related to missing or incomplete data. Although the same operative protocol and surgeons were utilized across both centers, inter-center variability may still influence outcomes. Furthermore, despite a considerable sample size for this type of surgical procedure, it may still be inadequate to discern subtle differences between groups or subgroups. Another limitation is the absence of specific genetic analyses; without detailed genetic characterization, the hereditary TAAA group may include heterogeneous patient populations with varying genetic risk profiles, potentially influencing clinical outcomes and complicating interpretation.

Despite these limitations, our study contributes to the ongoing discussion regarding the optimal management of TAAAs. The relative mortality and morbidity rates associated with open repair, as observed in our study, are considerable; also, performing and developing endovascular repair in young patients, and even those patients with hereditary aortopathies, is maybe warranted, except for those where an endovascular approach is not yet feasible.

## 5. Conclusions

Open TAAA repair in patients under 60 years old is related to a relevant perioperative mortality rate, with comparable outcomes in the hereditary and non-hereditary subgroups. Non-hereditary TAAA patients showed an impaired preoperative cardiopulmonary status.

## Figures and Tables

**Table 1:** Patient demographics: comparison of patients with and without hereditary thoracoabdominal aortic aneurysm.

		Overall	With Hereditary Aortopathy (n = 106)	Without Hereditary Aortopathy (n = 167)		
		Mean (Range)	Median (Range)	Median (Range)	*p*-Value	OR (95% CI)
Age, years		49 (14–59.99)	39.5 (18–59.69)	53.19 (14–59.99)	<0.001 *	0.85 (0.841–0.89)
BMI (kg × m^−2^)		24.89 (14.5–41.1)	22.84 (14.5–37.5)	25.99 (15.6–41.1)	<0.001 *	0.86 (0.79–0.92)
		N(%)	N (%)	N (%)		
Male		196 (71.79)	63 (59.43)	136 (79.64)	<0.001 *	0.35 (0.19–0.58)
Smoking		86 (31.50)	22 (20.75)	64 (38.32)	<0.01 *	0.42 (0.24–0.74)
ASA Score	<3	177 (64.84)	68 (64.15)	109 (65.27)	0.14	1.58 (0.76–3.25)
	≥3	35 (12.82)	17 (16.04)	18 (10.78)		
Diabetes mellitus		14 (5.13)	1 (0.94)	13 (7.78)	0.01 *	0.12 (0.01–0.66)
Any stage of kidney injury		94 (34.43)	16 (15.09)	78 (46.71)	<0.001 *	0.21 (0.11–0.37)
Hypertension		204 (74.73)	60 (56.60)	144 (86.23)	<0.001 *	0.21 (0.12–0.37)
Congestive heart failure		51 (18.68)	22 (20.75)	29 (17.37)	0.48	1.25 (0.67–2.31)
Coronary heart disease		44 (16.12)	5 (4.71)	39 (23.35)	<0.01 *	0.16 (0.06–0.41)
COPD		49 (17.95)	11 (10.38)	38 (22.75)	<0.01 *	0.40 (0.18–0.80)
Myocardial infarction		23 (8.42)	3 (1.10)	20 (11.98)	<0.01 *	0.22 (0.05–0.68)
PCI		47 (17.22)	14 (13.21)	33 (19.76)	0.16	0.62 (0.31–1.21)
CABG		13 (4.76)	5 (4.72)	8 (4.79)	0.97	0.99 (0.29–3.13)
Any type of aortic dissection		207 (75.82)	98 (92.45)	109 (65.27)	<0.001 *	6.38 (3.05–15.19)
Previous aortic surgery		160 (58.61)	81 (76.42)	79 (47.31)	<0.001 *	3.58 (2.10–6.25)

* *p* value < 0.05. Age and BMI comparisons were conducted via Mann–Whitney U test or Chi^2^ test. ASA: American Society of Anesthesiologists; BMI: body mass index; CABG: coronary artery bypass grafting; COPD: chronic obstructive pulmonary disease; PCI: percutaneous coronary intervention.

**Table 2:** Distribution of those with hereditary thoracoabdominal aortic aneurysm (%, n = 106).

Connective Tissue Disease	* N * (%)
Marfan Syndrome	90 (84.9)
Loeys–Dietz Syndrome	7 (6.6)
Ehlers–Danlos Syndrome	3 (2.8)
Other	6 (5.7)

**Table 3:** Perioperative comparison of patients with and without hereditary thoracoabdominal aortic aneurysm.

		Overall	With Hereditary Aortopathy	Without Hereditary Aortopathy		
Variables	Categories	* N * (%)	* N * (%)	* N * (%)	*p*-Value ^#^	OR (95% CI)
Emergency repair		37 (13.55)	10 (5.20)	9 (5.39)	0.15	0.35 (0.05–1.46)
Crawford classification	I	83 (30.40)	25 (23.58)	58 (34.73)	0.051	0.58 (0.33–1.00)
	II	89 (32.60)	42 (39.62)	47 (28.14)	0.90	1.67 (0.99–2.81)
	III	48 (17.58)	22 (20.75)	26 (15.57)	0.38	1.42 (0.75–2.67)
	IV	36 (13.19)	10 (9.43)	26 (15.57)	0.52	0.57 (0.25–1.21)
	V	17 (6.23)	7 (4.19)	10 (5.99)	0.15	1.11 (0.39–3.05)
Massive transfusion		131 (47.99)	52 (31.14)	79 (47.31)	0.77	1.07 (0.65–1.75)
Splenectomy		20 (7.33)	7 (4.19)	13 (7.78)	0.81	0.85 (0.30–2.16)
Surgery time (min)	Median (min–max)	419 (179–996)	433 (192–742)	404 (179–996)	0.51	1.00 (0.99–1.01)
Duration of stay (Hospital)	Median (min–max)	25 (1–247)	25.5 (1–247)	24.5 (1–131)	0.95	1.01 (0.99–1.02)
Duration of stay (IC)	Median (min–max)	9 (0–164)	9 (0–164)	10 (1–115)	0.99	1.00 (0.99–1.02)

^#^ Chi^2^ test.

**Table 4:** Morbidity: comparison of patients with and without hereditary thoracoabdominal aortic aneurysm for various complications.

		Overall	With Hereditary Aortopathy	Without Hereditary Aortopathy		
Variables	Categories	* N * (%)	* N * (%)	* N * (%)	*p*-Value	OR (95% CI)
In-hospital mortality		35 (12.82)	13 (12.26)	22 (13.17)	0.83	0.92 (0.43–1.91)
Wound complications		44 (16.12)	16 (15.1)	28 (16.77)	0.71	0.89 (0.44–1.72)
Pulmonary complications		173 (63.37)	71 (66.98)	102 (61.1)	0.32	1.29 (0.78–2.17)
	Pneumonia	126 (46.15)	46 (43.4)	80 (47.9)	0.47	0.83 (0.51–1.36)
	ARDS	31 (11.36)	16 (15.1)	15 (8.98)	0.12	1.80 (0.84–3.86)
Cardiac complications		63 (23.08)	24 (22.64)	39 (23.35)	0.89	0.96 (0.53–1.71)
	Myocardial infarction	4 (1.47)	1 (0.94)	3 (1.8)	0.57	0.57 (0.02–4.96)
	Atrial fibrillation	25 (9.16)	9 (8.49)	16 (9.58)	0.76	0.88 (0.36–2.05)
Acute kidney injury	Grade 1	40 (14.65)	12 (11.32)	28 (16.77)	0.21	0.64 (0.30–1.30)
	Grade 2	65 (23.81)	27 (25.47)	38 (22.75)	0.61	1.16 (0.65–2.05)
	Grade 3	7 (2.56)	3 (2.83)	4 (2.4)	0.82	1.20 (0.22–5.85)
Permanent hemodialysis		7 (2.56)	4 (3.77)	3 (1.8)	0.31	2.11 (0.43–11.66)
Neurological complications		87 (31.87)	30 (28.3)	57 (34.13)	0.31	0.76 (0.45–1.29)
	SCI	24 (8.79)	7 (6.6)	17 (10.18)	0.31	0.63 (0.23–1.54)
	Stroke	16 (5.86)	7 (6.6)	9 (5.39)	0.68	1.25 (0.43–3.50)
Bleeding complications		107 (39.19)	46 (43.4)	61 (36.53)	0.26	1.33 (0.81–2.19)
Sepsis		64 (23.44)	23 (21.7)	41 (24.55)	0.59	0.85 (0.47–1.52)
Reoperation during stay		96 (35.16)	40 (37.73)	56 (33.53)	0.49	1.20 (0.72–2.00)

ARDS: acute respiratory distress syndrome; SCI: spinal cord ischemia.

## Data Availability

Data are available on request.

## Supplementary Materials

The following supporting information can be downloaded at https://www.mdpi.com/article/10.3390/jcm14072513/s1, Tables S1–S3: Multivariable logistic regression analysis and subgroup analyses.
