# Supplementary material for: Outcome Following Open Repair of Hereditary and Non-Hereditary Thoracoabdominal Aortic Aneurysm in Patients Under 60 Years Old—A Multicenter Study
Source: J Clin Med. 2025 Apr 7;14(7):2513. doi: 10.3390/jcm14072513 (PMC11989434; doi:10.3390/jcm14072513)
Supplement: Supplementary file 1 [file jcm-14-02513-s001.zip › jcm-3550798-supplementary.pdf]

Supplementary Table S1. Multivariable logistic regression analysis of preoperative variables associated with in-hospital mortality.

Variable	Estimate	Std. Error	z value	P-value
**Intercept**	-0.0721	1.7053	-0.042	0.966
**Gender**	-0.0515	0.5557	-0.928	0.0353
**Smoking status**	0.0580	0.2254	0.257	0.797
**BMI**	-0.0817	0.0577	-1.416	0.157
**Any stage of Kidney injury**	0.4509	0.4734	0.952	0.341
**Diabetes mellitus**	0.0338	0.8916	0.038	0.970
**Hypertension**	0.5512	0.6093	0.905	0.366
**COPD**	-0.7432	0.5531	-1.344	0.179
**Coronary heart disease**	0.4675	0.5383	0.868	0.385
**Myocardial infarction**	0.2870	0.3959	0.725	0.469
**Previous aortic surgery**	0.6554	0.5008	1.309	0.191
**Any type of aortic dissection**	-0.4869	0.5264	-0.925	0.355

Supplementary Table S2. Subgroup analysis evaluating the impact of pre-existing renal insufficiency on postoperative acute kidney injury in hereditary versus non-hereditary TAAA patients.

Variable	Overall (N=94 - %)	With hereditary aortopathy (N=16 - %)	Without hereditary aortopathy (78 - %)	P-value	OR (95% CI)
**Postoperative acute kidney injury**	6 (6.38)	0 (0%)	6 (7.69)	0.55	0 (0 – 4.24)

Supplementary Table S3. Subgroup analysis evaluating the impact of previous aortic surgery on postoperative mortality and morbidity in hereditary versus non-hereditary TAAA patients.

Variable	Overall (N=160 - %)	With hereditary aortopathy (N=81 - %)	Without hereditary aortopathy (N=79 - %)	P-value	OR (95% CI)
**In-hospital mortality**	22 (13.75)	11 (13.58)	11 (13.92)	1	0.97 (0.36 – 2.65)
**Re-operation during stay**	64 (40)	34 (41.98)	30 (37.97)	0.72	1.18 (0.60 – 2.34)

**Bleeding complications**	76 (47.5)	41 (50.62)	35 (44.30)	0.52	1.29 (0.66 – 2.52)
**Neurological complications**	52 (32.5)	23 (28.40)	29 (36.71)	0.34	0.69 (0.33 – 1.40)
